# Tongue coating, severity of *Ama*, and disease activity in patients with Rheumatoid Arthritis: A pilot study

**DOI:** 10.1016/j.jaim.2025.101315

**Published:** 2026-05-16

**Authors:** Shrikant Wagh, Vinayak Joshi, Ashwinikumar A. Raut, Sanjeev Rastogi, Digambar Deepankar, Srikanth Babu Perugu, Madhavi Mahajan

**Affiliations:** a Dept of Rognidan & Vikruti-vigyan, Dr D Y Patil College of Ayurveda and Research Centre, Dr D Y Patil Vidyapeeth (Deemed to Be University), Pune, India; b Clinical Research and Integrative Medicine, Kasturba Health Society – Medical Research Centre, Mumbai, India; c Department of Integrative Medicine, Vatsala Hospital, Lucknow, India; d Ayurveda-Arthritis Treatment and Advanced Research Centre (A-ATARC), India; e Annals of Ayurvedic Medicine, India; f Dept of Kayachikitsa, Dr D Y Patil College of Ayurveda and Research Centre, Dr D Y Patil Vidyapeeth (Deemed to Be University), Pune, India; g Dept of Kayachikitsa, Dr BRKR Govt Ayurveda College, Hyderabad, India; h Dept of Kayachikitsa, BVDU College of Ayurveda, Pune, India

**Keywords:** *Ama*-score, Clinical disease activity index, *Jivha-samata*, Smartphone tongue image, Winkel's tongue coating index

## Abstract

**Background:**

Tongue examination has been documented in Ayurveda and other medical systems. The severity of tongue coating (TC), an indicator of *Ama* in Ayurveda, can be clinically assessed by the Winkel tongue coating index (WTCI) through the analysis of smartphone images. This pilot study examines the relationship between TC and disease severity, as well as TC and *Ama* severity, in patients with Rheumatoid Arthritis (RA), a prototype of Amavata.

**Materials and methods:**

Consecutive outpatients suffering from RA (diagnosed as per ACR/EULAR criteria, 2010) were divided into two groups- Group A: Treatment naïve patients with active disease, and Group B: patients on treatment in remission as determined by Clinical Disease Activity Index (CDAI). The *Ama*-severity in these subjects was assessed by a validated *Ama*-instrument. A photograph of the tongue obtained by a smartphone was selected, edited, and marked into sextants for WTCI assessment. These photographs were randomized and submitted for TC analysis to five expert clinicians. Eleven images in each group could be analysed by all assessors. The clinical records of the respective patients were used for statistical analysis (GraphPad InStat Version 3.6 software).

**Results:**

Twenty-two cases, 11 with active, untreated RA (Group A), and 11 in remission (Group B), were studied. All patients were females except one. Patients in Group A were older, less educated, and had a lower BMI than those in Group B. There was moderate inter-rater agreement (0.76 in Group A and 0.73 in Group B). There was no significant correlation between CDAI and WTCI (Spearman r = −0.08356, *P* = 0.7116, 95 % CI = −0.4981 - 0.3621) as well as between *Ama* Score and WTCI (Spearman r = - 0.1608, *P* = 0.4748, 95 % CI = −0.5548 - 0.2921). However, the Bonferroni correction indicated a statistically significant association between WTCI and the *Ama*-score. Moreover, the Friedman test revealed maximum coating in the posterior-middle area (area B, Fr = 57.728; *P* = < 0.0001, 95 % CI 1.645 to 1.937). Also, a moderately positive correlation between the TC score in area B and *Ama-*score (Spearman r = 0.4790, *P* = 0.0241, 95 % CI = 0.05858–0.7551) as well as between TC Score in area B and CDAI (r = 0.4393, *P* = 0.0408, 95 % CI = 0.008332 to 0.7327) was observed.

**Conclusion:**

This pilot study indicates that there is a positive association between WTCI and *Ama*-score in *Amavata* (RA). A moderately positive correlation between the severity of TC in the posterior-middle area and the severity of *Ama* in *Amavata* (RA) implies a careful examination of the posterior tongue in these patients. The inter-observer reliability was moderate during this study. Studies with a larger sample size are recommended based on observations of this study.

## 1 Introduction

Tongue examination is an integral part of patient assessment in every healthcare system, modern or traditional. Tongue coating (TC) is an important aspect of tongue examination. Quantification of TC during visual inspection has been attempted in the form of grades (no coating to severe coating), area involved, colour of the coating, or wet-weight of the tongue scraping [1]. The Winkel Tongue Coating Index (WTCI) divides the tongue into six parts (sextants), three posterior and three anterior. Each part is labelled as no coating (Grade 0), light coating (Grade 1, pink colour observed underneath the coating), and severe coating (Grade 2, pink colour not visible underneath the coating) [2]. The maximum WTCI is thus 12. Shimizu et al. scored the coating as grade 0 (no coating), grade 1 (thin, visible papillae underneath the coating), and grade 2 (thick, papillae not visible) [3]. Though the number of divisions of the tongue differs in these two indices, the methods of assessment appear to be complementary to each other. Tongue assessment can also be carried out from images by trained and experienced personnel. TC assessment can be based on images obtained with a smartphone if a standard procedure is followed for taking the photos, viz., camera settings, light source, and display [4].

In Ayurveda, tongue examination is described as a component of the eight-fold clinical examination [5]. Ayurveda clinicians consider TC as an important indicator of *Ama* (improperly digested food, accumulated waste products, or toxic complexes). The disease activity of *Amavata* (Rheumatoid Arthritis) can be assessed by a validated *Ama* assessment instrument (AAI), rated by the patient on a 0–10 visual analogue scale [6].

Rheumatoid arthritis (RA), the most common form of inflammatory arthritis, is a prototype of *Amavata*. It is classified according to the 2010 ACR/EULAR criteria [7], and its activity can be measured with recommended instruments such as the Clinical Disease Activity Index (CDAI) [8]. There are no studies that establish a correlation between the degree of TC, an indicator of *Ama*, and disease activity of *Amavata* using AAI, and that of RA determined by CDAI. It is, therefore, necessary to study such a correlation in view of its importance and potential impact on the diagnosis, assessment, and management of patients with *Amavata* (RA).

The primary objective of this pilot study was, therefore, to evaluate the correlation between the severity of TC based on analysis of smartphone-captured images and CDAI. The secondary objectives were to evaluate the correlation between AAI and the severity of TC, and to identify inter-rater reliability in the assessment of TC in these patients.

## 2 Material and methods

This cross-sectional pilot study was carried out at Lupus Clinic, a super-specialty Rheumatology outpatient clinic in Pune, during September 2024 and December 2024. Ethical clearance was issued by the Institutional Ethics Committee of Dr. D. Y. Patil College of Ayurveda and Research Centre, Pune, vide letter number DYPCARC/IEC/662 B/2023 dated September 20, 2023.

**Inclusion criteria:** Patients of RA above 18 years of age and willing to sign consent were included in the study. Two groups of patients were registered for assessment of TC:

**Group A:** Consecutive treatment-*naïve* patients of RA (*Amavata*) diagnosed as per 2010 ACR/EULAR classification criteria.

**Group B:** Consecutive patients of RA previously diagnosed as per 2010 ACR/EULAR classification criteria who are now in remission (CDAI <2.2) following successful treatment for variable periods with disease modifying anti-rheumatic drugs (DMARDs).

**Exclusion criteria:** The following patients were excluded:

1. Pregnant and lactating females
2. Joint pain and swelling following trauma
3. Patients with fever (axillary temperature more than 99^0^ F)
4. Neurological disorders that limit mobility, e.g., hemiplegia, paraplegia, Parkinsonism, lumbar canal stenosis, etc.
5. Uncontrolled endocrine disorders, e.g., diabetes mellitus, hypothyroidism
6. History of chronic gastrointestinal disorder, e.g., GERD, inflammatory bowel disease
7. Subjects using the following drugs were excluded:
  1) Group A:
    i) DMARDs or long-acting glucocorticoids (GC) during the previous three months
    ii) Nonsteroidal anti-inflammatory drugs (NSAIDs) or GCs within the previous 24 h of examination
  2) Group B: Nonsteroidal anti-inflammatory drugs or glucocorticoids within the previous 24 h of examination

General and systemic examination, joint counts and CDAI estimation, and *Ama*-score assessment using AAI were carried out in all patients.

**Fig. 1:**
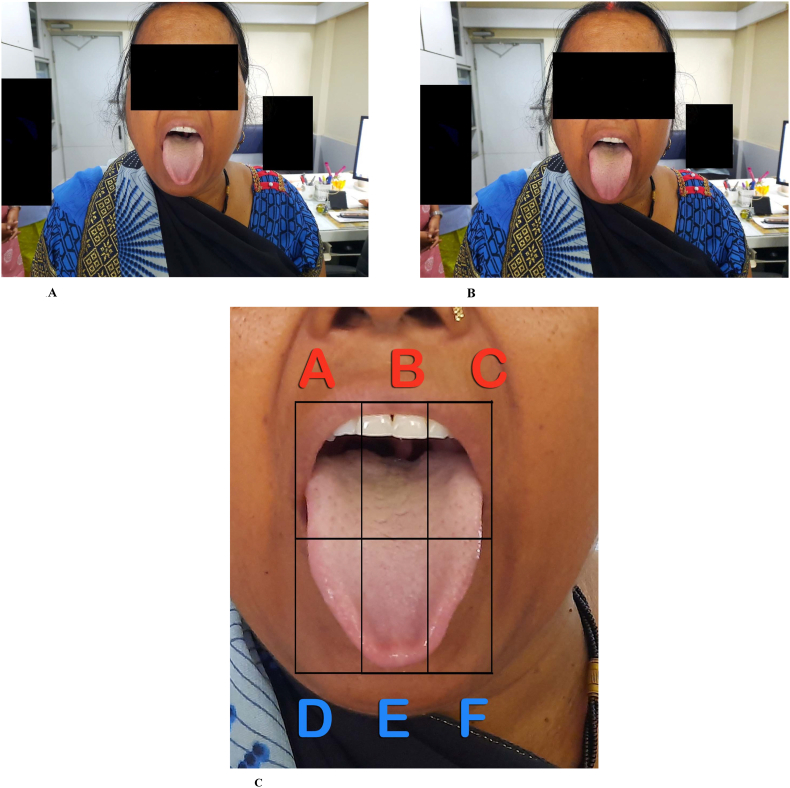
Extraction of tongue image from original photos A and B are original photos, and C is the extracted image.

**Fig. 2:**
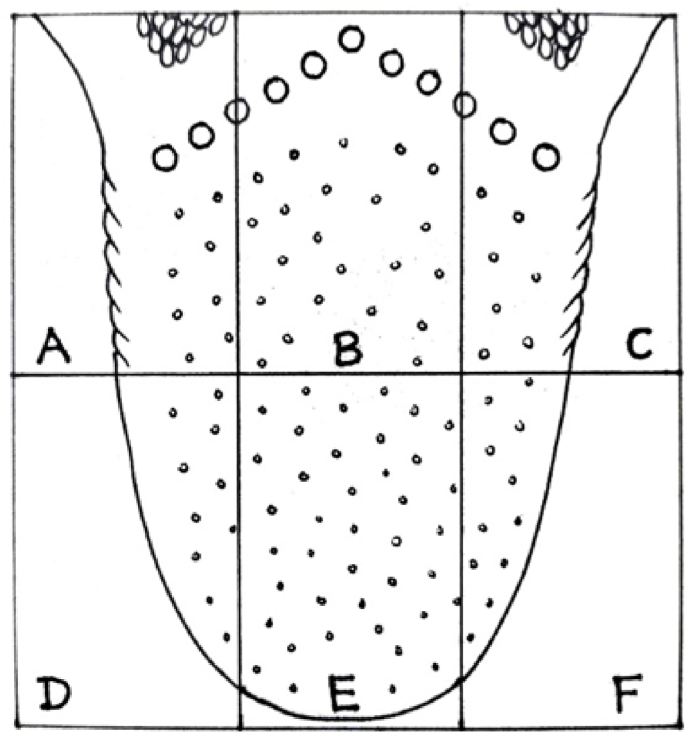
Winkel tongue coating index.

**Fig. 3:**
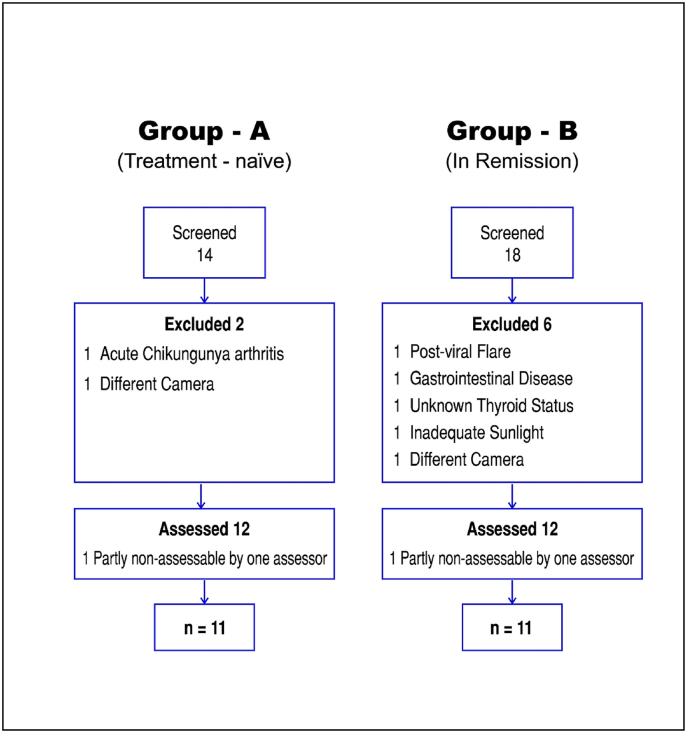
Study flow chart.

**Fig. 4:**
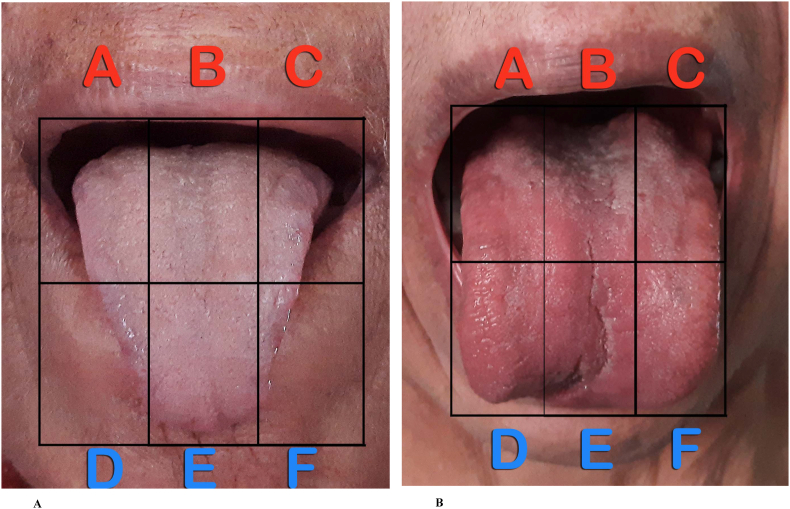
Illustrative cases with WTCI, CDAI, and *Ama*-scores in Groups A (Active) and B (Remission) WTCI = Winkel's Tongue Coating Index, CDAI = Clinical Disease Activity Index in Rheumatoid arthritis.

Two photographs of the tongue were obtained in each patient in the following manner: The subject was asked to do plain-water gargles 5 min before the photo shoot. S/he was asked to say ‘aah’ (to elevate the soft palate) and protrude the tongue naturally. The photos were clicked in daylight from 20 to 25 cm distance by a single investigator (SW) using a Samsung Galaxy 50s smartphone camera (48 megapixel). Both photographs were transferred to the computer via email. One of these two images was selected. The criteria for selection of an image were: 1) an adequately open mouth, 2) clarity of the details of the tongue, and 3) a complete view of the tongue. This image was cut into sextants and labelled (A-F areas) without affecting its quality by a professional photographer (Fig. 1). Twenty-four photos (12 from each group) were randomly numbered and submitted for assessment to five assessors (DD, MM, SP, SR, and AR). The assessors were asked to score (0–2) each area for TC. They were allowed to use the Winkel (pink colour underneath coating) and/or Shimizu (visible papillae underneath coating) method for the assessment of TC in each sextant (Fig. 2, Table 1). One of the assessors was unable to assess few sextants in one image from each group. These images were not considered for the study. Thus, scores of 11 images from each group and the respective patients were included in the study (Fig. 3). These TC scores from all assessors and the corresponding patient data, viz. ama score and CDAI (Fig. 4), were processed at the central computer and submitted for statistical analysis. GraphPad InStat Version 3.6 (www.graphpad.com) software was used for statistical analysis of data. P value < 0.05 was considered significant. Spearman's Rank Correlation was used for correlation between variables (for non-normal distribution). Mann-Whitney *U* Test was used for inter-group comparison (for non-normal distribution).

**Table 1:** Parameters used for Winkel Tongue Coating Index.

Score	Winkel	Shimizu
**0 = None**	No visible coating	No visible coating
**1 = Thin**	Pink colour is seen underneath the coating	Papillae visible
**2 = Thick**	Pink colour is not seen underneath the coating	Papillae not visible

## 3 Results

Twenty-two cases, 11 with active, untreated RA and 11 in remission, were studied. There was only one male patient in Group A. All others were females. Patients in Group A were older, less educated, and had a lower BMI than those of Group B (Table 2).

**Table 2:** Characteristics of the patients.

	Group A (Active) n = 11	Group B (Remission) n = 11
**Age Years (Mean, SD)**	52.72 ± 13.02	47.36 ± 10.02
**Male/Female**	1/11	0/11
**Education**	Uneducated = 4 Up to 10th standard = 5 College studies = 2	Uneducated = 2 Up to 12th standard = 6 Degree/Postgrad = 3
**Occupation**	Homemaker (8) Farmwork (2) Shop-owner (1)	Homemaker (10) Farmwork (1)
**BMI (Mean, SD)**	21.76 ± 5.12	24.22 ± 2.79

The average inter-rater variability (% agreement) in active cases (Group A) and cases in remission (Group B) was 0.76 (moderate) and 0.73 (moderate), respectively. The kappa value suggests moderate inter-rater agreement in both groups.

The median values of the *Ama*-score in Group A and Group B were 35 (12–76.5) and 04 (00–22), respectively. On inter-group comparison, the difference between the *Ama*-score of the two groups was statistically significant (p < 0.001). The median values of CDAI Score in Group A and Group B were 29 (24–39.5) and 00 (00–02), respectively. On inter-group comparison, the difference between the CDAI of the two groups was statistically significant (p < 0.001). The median values of WTCI Score in Group A and Group B were 08 (4.6–9.6) and 6.4 (2.6–10.6), respectively. The difference between the CDAI of the two groups was statistically not significant (p = 0.5543) on inter-group comparison (Table 3).

**Table 3:** Inter-group comparison.

	Ama Score		CDAI		WTCI	
	Group A	Group B	Group A	Group B	Group A	Group B
**Sample Size (n)**	11	11	11	11	11	11
**Mean ± SD**	38.77 ± 20.37	6.73 ± 7.46	30.77 ± 5.37	0.50 ± 0.67	7.65 ± 1.55	6.98 ± 2.44
**95 % CI**	25.09–52.46	1.72–11.74	27.16–34.38	0.05–0.95	6.61–8.70	5.34–8.62
**Median (Range)**	35 (12–76.5)	04 (00–22)	29 (24–39.5)	00 (00–02)	08 (4.6–9.6)	6.4 (2.6–10.6)
**Inter-Group Comparison**	Mann–Whitney test		Mann–Whitney test		Mann–Whitney test	
**Mann–Whitney U statistic**	5.000		0.000		51.000	
**U’**	116.00		121.00		70.000	
P value	<0.0001, statistically significant		<0.0001, statistically significant		0.5543, statistically not significant	

**Fig. 5:**
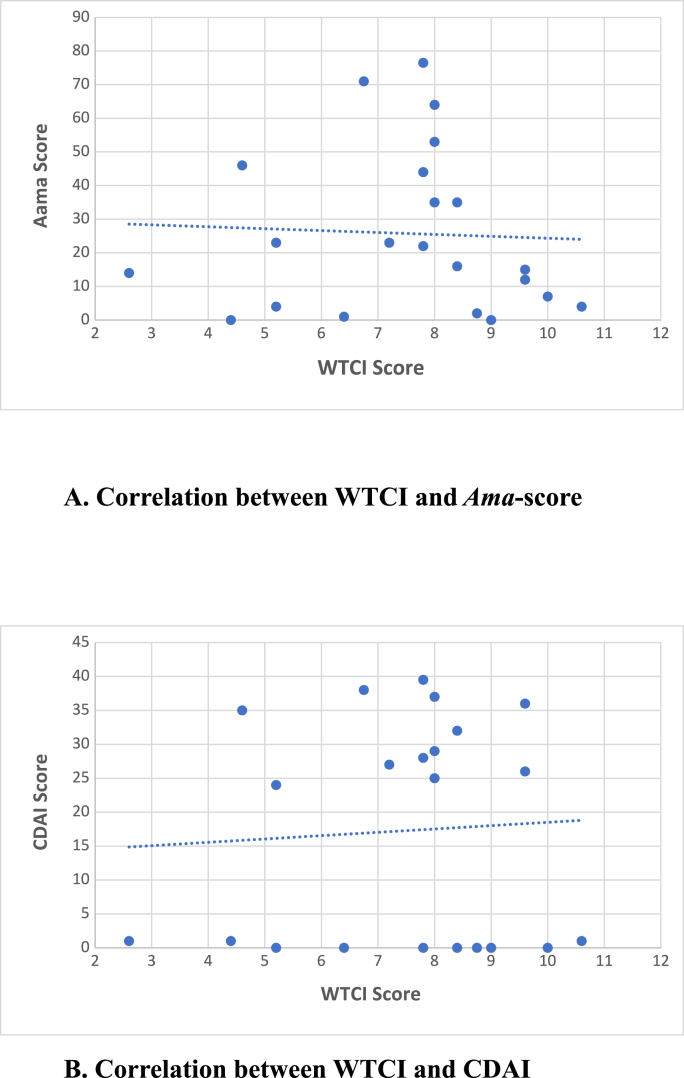
Correlation between WTCI, *Ama* score, and CDAI. WTCI = Winkel's Tongue Coating Index, CDAI = Clinical Disease Activity Index in Rheumatoid arthritis.

Spearman's rank correlation was computed to assess the relationship between the severity of TC (assessed by WTCI) and the severity of Ama (evaluated by AAI). There was a negative correlation between WTCI Score and Aam Score, r = - 0.1608 (corrected for ties), P = 0.4748 (95 % CI = −0.5548 to 0.2921). The P value is not significant (Table 4, Fig. 5).

**Table 4:** Correlation between Ama score, CDAI, and WTCI.

22 Subjects	Median (Range)	Spearman r (Corrected for ties)	95 % CI	*P* (two-tailed)
**WTCI**	7.9 (2.6–10.6)	−0.1608	−0.5548 to 0.2921	0.4748
**Ama Score**	19 (0–76.5)			
**WTCI**	7.9 (2.6–10.6)	−0.08356	−0.4981 to 0.3621	0.7116
**CDAI**	24.5 (0–39.5)

Spearman's rank correlation was also calculated to assess the relationship between the severity of TC (assessed by WTCI) and disease activity of RA (evaluated by CDAI). There was a negative correlation between WTCI Score and CDAI, r = −0.08356 (corrected for ties), P = 0.7116 (95 % CI = −0.4981 to 0.3621). The P value is not significant (Table 4, Fig. 5).

The statistical significance of differences in TC scores between clinical groups was analysed to reduce the risk of Type I error due to multiple testing. Multiple pairwise comparisons were performed using the Bonferroni correction for control of the family-wise error rate. Three pairwise comparisons were made: WTCI with Ama-score, WTCI with CDAI, and Ama-score with CDAI. Based on the Bonferroni-adjusted p-values, only the comparison between WTCI and Ama-score remained statistically significant (P = 0.0111, 95 % CI -27.72 to −3.141), suggesting a robust association between Ama-score and TC. The remaining comparisons did not show significant associations.

TC in each of the sextants was assessed to appreciate the pattern of coating. Friedman's test revealed significant differences in the areas coated. The medians indicated that there was maximum TC in area B (posterior-middle, Med = 2.00), followed by area A (posterior-right), area C (posterior-left), and area E (anterior-middle), Fr = 57.728 (corrected for ties), P = < 0.0001 (95 % CI 1.645 to 1.937). This P value is significant (Table 5).

**Table 5:** Area-wise assessment of WTCI.

	Area A	Area B	Area C	Area D	Area E	Area F
**Sample Size (n)**	22	**22**	22	22	22	22
**Mean ± SD**	1.21 ± 0.36	**1.79** ± **0.33**	1.32 ± 0.45	0.9 ± 0.47	0.60 ± 1.20	0.93 ± 0.45
**Median (Range)**	1.2 (0.4–2.0)	**2.0 (1.0–2.0)**	1.2 (0.3–2.0)	1.0 (0.0–1.8)	1.2 (0.0–2.0)	1.0 (0.0–1.6)
**Sum of Ranks**	82.50	**124.00**	88.00	47.00	71.00	49.50

Friedman Statistic Fr = 57.728 (corrected for ties), P < 0.0001 (95 % CI 1.645 to 1.937).

**Fig. 6:**
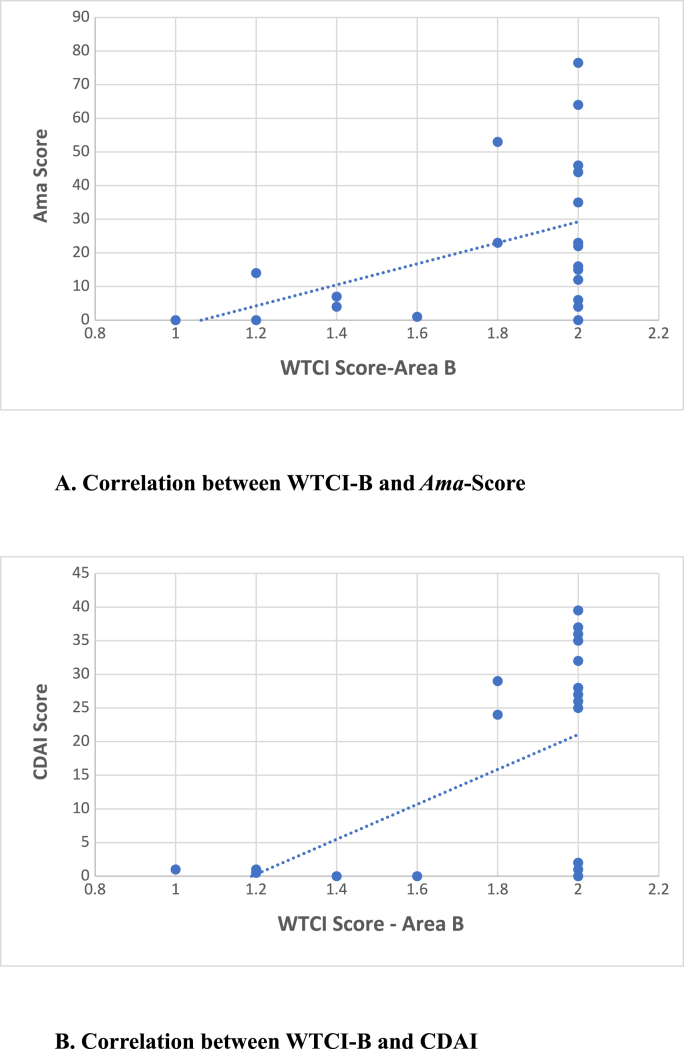
Correlation between Ama score, CDAI, and WTCI (Area B = middle-posterior) WTCI = Winkel's Tongue Coating Index, CDAI = Clinical Disease Activity Index in Rheumatoid arthritis.

Spearman's rank correlation was computed to assess the relationship between the severity of TC in area B (posterior-middle), assessed by WTCI, and the severity of Ama (evaluated by AAI). There was a moderately positive correlation between the TC score in area B and Aam Score, r = 0.4790 (corrected for ties), P = 0.0241 (95 % CI = 0.05858 to 0.7551). The P value is considered significant (Table 6, Fig. 6).

**Table 6:** Correlation between Ama score, CDAI, and WTCI-Area B (Posterior-Middle).

22 Subjects	Median (Range)	Spearman r (Corrected for ties)	95 % CI	*P* (two-tailed)
**WTCI- B**	2.0 (1.0–2.0)	0.4790	0.05858 to 0.7551	0.0241
**Ama Score**	19 (0–76.5)			
**WTCI- B**	2.0 (1.0–2.0)	0.4393	0.008332 to 0.7327	0.0408
**CDAI**	24.5 (0–39.5)

Spearman's rank correlation was also calculated to assess the relationship between the severity of TC in area B (posterior-middle) assessed by WTCI and disease activity of RA (evaluated by CDAI). There was a moderately positive correlation between TC Score in area B and CDAI, r = 0.4393 (corrected for ties), P = 0.0408 (95 % CI = 0.008332 to 0.7327). The P value is considered significant (Table 6, Fig. 6).

## 4 Discussion

This is the first-ever study reported that objectively examines the tongue for coating in an *Ama*-related Ayurveda disorder. viz. *Amavata* (RA). The results of this pilot study indicate that there is a good correlation between smartphone captured images of TC and the *Ama*-score assessed by AAI in patients with *Amavata* (RA). Maximum TC is observed in the posterior-middle area of the tongue in these patients. However, the WTCI did not correlate with CDAI. The inter-rater variability (5 assessors) was moderate (76 % in group A, and 73 % in group B, respectively) in these subjects.

There is no reference to TC in the three classical treatises of Ayurveda, except as a prodromal feature of *Prameha.* [9] However, the tongue is commonly examined by Ayurveda physicians as an indicator of *Ama*-dominance during clinical evaluation. The tongue being a site of *Kapha*, it is logical to undertake tongue examination in patients of *Amavata*.

The CDAI is a commonly used, validated composite index used to assess disease activity in RA. It evaluates 28 joints (hands, wrists, elbows, shoulders, and knees) for swelling and tenderness. The global disease activity as assessed by the patient (“Considering all the ways arthritis affects you, how well are you doing?“) and the assessor (VAS 0–10), is also included. CDAI is a simple sum of swollen joint count, tender joint count, and global disease activity as assessed by the patient and the physician [10]. Although the degree of coating did not correlate with CDAI, it is interesting to note that a robust correlation is observed between TC and the *Ama*-score after applying the Bonferroni correction. Moreover, there is a moderately positive correlation between the severity of TC in the posterior-middle area of the tongue and the severity of *Ama* in *Amavata* (RA), as well as the severity of TC in this area and the degree of disease activity in RA, assessed by CDAI. *Ama* is formed in the intestines in the form of improperly digested food and/or accumulated waste products. It is noteworthy that the posterior tongue represents the intestines in TCM. Some anterior spill-over of excessive ama may occur in *Amavata* (RA), and thus, the posterior-middle area gets coated. Anterior extension of TC from the root of the tongue and lateral extension from the median line are graded as more severe in TCM tongue diagnosis [11].

There are various methods to assess TC [12]. Winkel's Index was selected for this study because of its wide usage and easy applicability. Smartphone captured tongue images are acceptable, as evident from mobile apps being developed for self-monitoring of health by people at large. The WTCI is itself meant for the analysis of images of the tongue. Conventional inspection of the tongue relies on the knowledge and experience of the examiner. In our study, all assessors were senior and respected Ayurveda clinicians from different regions of the country. They were provided with appropriate information regarding the scoring system. The method of capturing the image was also standardized. Use of a single smartphone camera, clicking by a single photographer, day light, and a fixed distance from which the photo was clicked were all predefined and rigorously followed. Computer-aided image enhancement [13], tongue diagnosis systems, image and pattern recognition with artificial intelligence [14] are in preliminary stages and may not be useful in routine clinical practice.

*Ama* originates in the gut. There is about 45 % similarity in the oral and intestinal microbiota. The microbiota of the tongue and gut may have similar metabolic regulatory mechanisms. The intestinal microbiota and TC microbiota both exhibit changes in abundance when immune receptors are activated. Studies on arthritis in animal models have demonstrated a link between gut and oral microbiota and joint inflammation. Patients with RA have a relative abundance of proinflammatory microbial species such as *Prevotella* and *Lactobacilli*. Alterations in these microbiomes are implicated in the loss of tolerance against self-antigens and during increased inflammatory events such as arthritis [12]. TC-microbiota vary significantly during active and quiescent phases of RA as compared to healthy individuals [13]. Thus, TC-microbiota form an ideal research area because they can be easily collected and have a moderate rate of renewal. Evaluation of TC for *Ama* is, thus, a preliminary clinical examination that is critical in *Ama*-related disorders.

There are certain limitations to this study. This was a pilot study with a few patients wherein we standardized the procedure for taking the photos and formulated operational definitions and objective methodology for assessment of TC in *Amavata* (RA). These measures are likely to improve the reliability of this small study. There was 76 % (Group A) and 73 % (Group B) inter-rater agreement for the assessment of TC in this study. It has been reported that the reliability of Ayurveda diagnosis, including tongue diagnosis, is poor. The tongue examination was carried out in 20 healthy individuals by 15 registered Ayurvedic doctors with 3–15 years of experience, independently in 20 healthy subjects. Weighted kappa statistics for tongue examination were slight to fair for all data sets. The average kappa for tongue was 0.17 (0.00–41.00) only [15]. The assessors in this study are very senior, and the tongue was assessed in diseased subjects. This may be a reason for a much better inter-rater agreement in this study. Digital tongue imaging systems with computer-aided analysis can be used for further research in this area.

The number of subjects studied in this pilot study is small and may appear inadequate. However, most of the reported studies in this area have analysed up to 30 images, as a higher number of images can lead to assessor exhaustion and inaccurate assessments. All assessors reported the image quality to be good and adequate, except for two images that could not be evaluated by one out of five assessors. One assessor expressed concern about differentiating a pale tongue from a coated tongue, though none of these patients was severely anaemic. Another assessor felt that the assessment of a fissured tongue for coating was somewhat challenging. It is suggested that differentiation between physiological keratinization and bacterial biofilm is a major cause of inter-rater variability. Bacterial biofluorescence can visualise TC microflora and quantify the biofilm characteristics [16]. However, this method may not be applicable in routine clinical practice.

An incompatible (*Viruddha*) diet is an important cause of the formation of *Ama* [17]. Dietetic and other factors were not investigated in this pilot study. Diseases like chronic gastritis, pancreatitis, metabolic syndrome, chronic kidney disease, and breast cancer are associated with increased TC [18]. The oral microbiomes in TC are significantly higher in patients with active RA than in those with inactive RA [19]. The gut permeability is altered in patients with RA, as indicated by serum markers [20]. *Ama* may be a representative of the gut microflora and permeability. Therefore, proper examination of the posterior tongue is an invaluable tool for the assessment of *Ama* in clinical practice.

## 5 Conclusion

This is the first ever reported pilot study that attempts to correlate the degree of TC with the severity of *Ama* in *Amavata* and disease activity in RA. There is a robust association between the *Ama*-score and TC. There is a moderately positive correlation between the severity of TC in the posterior-middle area and the severity of *Ama,* as well as the disease activity of RA. This study implies that the *Ama*-status in *Amavata* (RA) can be assessed to a certain extent by examination of the posterior tongue. Studies with a larger sample size are necessary, considering the pilot status of this work. In the current era of telemedicine, tongue examination can be extremely helpful. Therefore, similar studies need to be undertaken for other *Ama*-related diseases such as *Grahani* (chronic colitis)*, Ama-atisar* (initial phase of diarrhoea)*, Visuchika* (cholera-like watery diarrhoea), and *Ama-jwara* (initial phase of fever). Studies with a larger sample size are desirable for further validation of these observations.

## Data statement

The study data can be obtained from the corresponding author upon a reasonable academic request.

## CRediT author statement

SW: Conceptualization, methodology, investigation, resources, data curation, writing the initial draft.

VJ: Guidance, supervision.

AR: Assessment of images, writing-review.

SR: Assessment of images, writing-review.

DD: Assessment of images.

SP: Assessment of images.

MM: Assessment of images.

## Use of generative AI in scientific writing

None.

## Funding sources

None.

## Declaration of competing interest

The authors declare that they have no known competing financial interests or personal relationships that could have appeared to influence the work reported in this paper.
